# Oocyst-Derived Extract of *Toxoplasma Gondii* Serves as Potent Immunomodulator in a Mouse Model of Birch Pollen Allergy

**DOI:** 10.1371/journal.pone.0155081

**Published:** 2016-05-05

**Authors:** Angelika Wagner, Irma Schabussova, Mirjana Drinic, Johnnie Akgün, Gerhard Loupal, Michael Kundi, Anja Joachim, Ursula Wiedermann

**Affiliations:** 1 Institute of Specific Prophylaxis and Tropical Medicine, Center for Pathophysiology, Infectiology & Immunology, Medical University of Vienna, Vienna, Austria; 2 Institute of Pathology and Forensic Veterinary Medicine, Department of Pathobiology, University of Veterinary Medicine, Vienna, Austria; 3 Institute of Environmental Health, Center for Public Health, Medical University of Vienna, Vienna, Austria; 4 Department of Pathobiology, Institute of Parasitology, University of Veterinary Medicine, Vienna, Austria; French National Centre for Scientific Research, FRANCE

## Abstract

**Introduction:**

Previously, we have shown that oral infection with *Toxoplasma gondii* oocysts prevented type I allergy in mice. Here we investigated whether the application of a *T*. *gondii* oocyst lysate antigen (OLA) could also reduce allergy development. BALB/c mice were immunised twice with OLA followed by sensitisation with the major birch pollen (BP) allergen Bet v 1 and an aerosol challenge with BP extract.

**Methods:**

First, we tested OLA *in vitro*. Stimulation of splenocytes and bone marrow-derived dendritic cells (BMDC) with OLA led to the production of pro-inflammatory and regulatory cytokines such as IL-6, IFN-γ and IL-10. Moreover, BMDC exposed to OLA upregulated the maturation markers CD40, CD80, CD86, and MHCII. Furthermore, OLA was recognised by TLR2-transfected human embryonic kidney cells.

**Results:**

Immunisation of mice with OLA induced high levels of *Toxoplasma*-specific IgG antibodies in sera along with increased production of IFN-γ and IL-10 in *Toxoplasma*-antigen restimulated splenocytes. OLA reduced allergic airway inflammation as manifested by significant reduction of eosinophils in bronchoalveolar fluids, decreased cellular infiltrates and mucus production in the lungs. Accordingly, Bet v 1-specific IgE was decreased in OLA-pretreated mice. The reduced allergic immune responses were accompanied by increased numbers of CD4^+^CD25^high^Foxp3^+^ regulatory T cells in spleens as well as by increased numbers of granulocytic myeloid-derived suppressor cells in lungs when compared to sensitised controls suggesting that these two cell populations might be involved in the suppression of the allergic immune responses.

**Conclusion:**

Our data demonstrate that pretreatment with the oocyst extract can exert anti-allergic effects comparable to *T*. *gondii* infection. Thus, the immunomodulatory properties of the parasite extract indicate that this extract and in the future defined molecules thereof might serve as immunomodulatory adjuvants in allergy treatment and prophylaxis.

## Introduction

Within the last decades allergic diseases have been increasing in prevalence in industrialised countries and recently also in urban areas of developing countries [1, 2]. Factors associated with the altered epidemiology of allergic diseases include changes in lifestyle, pollution and diet, as well as lack of exposure to certain infectious agents including helminths and protozoa such as *Toxoplasma gondii* [3].

The ubiquitous intracellular protozoon *T*. *gondii* is frequently transmitted by the faecal-oral route [4] when ingesting contaminated food or water. Currently the prevalence in Northern Europe and North America varies between 10–30% as opposed to seroprevalence of more than 50% in South America and Africa [5]. In the majority of human cases the infection remains asymptomatic but can be severe in immunocompromised patients and foetuses [6]. Murine studies demonstrated that acute infection with *T*. *gondii* initiates a strong Th1-polarised immune response, predominantly orchestrated by IFN-γ, limiting parasite replication and potentially leading to its destruction [7]. In order to prevent Th1-driven immunopathology, a counter-balancing cytokine response, mainly via IL-10, is required [8]. The initiation of both inflammatory and regulatory immune responses make *T*. *gondii* a highly attractive candidate to study immunomodulation.

The observation that reduced exposure to certain microbes early in childhood might be associated with an increased risk of developing allergic diseases later in life led to the formulation of the hygiene hypothesis [9]. Epidemiologic data further indicated that seropositivity for *T*. *gondii* is inversely related to allergic sensitisation and symptomatic manifestation of allergies in humans [10–15]. In order to study the immunomodulatory properties of this parasite and its impact on allergy development, we previously established a mouse model of *T*. *gondii* infection [16]. In this model we observed that the infection is bi-phasic with innate and Th1- mediated immune responses (TLR activation and IFN-γ, IL-6 and TNF-α production) during the acute phase of infection and an increasing systemic regulatory immune response (rising frequency of regulatory T cells as well as increased levels of IL-10 and TGF-β) in the chronic phase. Furthermore, we have shown that *T*. *gondii* infection suppressed Th2-related inflammation in a mouse model of birch-pollen-induced allergic airway inflammation [16]. These results were confirmed by Fenoy et al. [17, 18] further describing a major role for regulatory T cells in thoracic lymph nodes of *T*. *gondii*-infected mice in preventing allergic lung inflammation in their model.

Even though infections with parasites showed beneficial effects on immune-mediated inflammatory diseases, the use of live parasites might lead to severe unpredictable side effects [19]. Consequently, the use of inactivated parasites or parasite-derived molecules thereof seems to be a more appropriate strategy for prevention and treatment, provided that these extracts/molecules elicit comparable immune-modulatory properties as the live parasite. So far different parasite extracts derived from helminths have been tested in mice for their capacity to suppress allergic immune responses [20–24]. With respect to intracellular parasites, such as the protozoan *T*. *gondii*, neither inactivated parasites nor extracts have been tested for their anti-allergic effects so far.

The aim of this study was therefore to evaluate whether modulation of allergic responses depends on the infection with live *T*. *gondii* oocysts or can be achieved with the lysate antigen extract prepared from the sporozoite-containing *T*. *gondii* oocysts (OLA) antigens. Following *in vitro* testing of OLA, this extract was applied in our clinically relevant mouse model of birch pollen allergy (BP) to evaluate its immunomodulatory potential to down-modulate allergen-specific immune responses.

## Materials and Methods

### Mice

Female BALB/c mice (n = 4–8 per group) aged 6–8 weeks were purchased at the Research Institute for Laboratory Animal Breeding at the Department of Biomedical Research, Medical University Vienna (Himberg, Austria). BALB/c mice were housed in individually ventilated cages and provided with food and water ad libitum at the Department of Biomedical Research, Medical University of Vienna. Experiments were approved by the Animal Experimentation Committee of the Medical University of Vienna and the Austrian Federal Ministry of Science and Research.

### Parasites lysate antigens

*T*. *gondii* oocysts (laboratory strain “Hannover 1”) and tachyzoites (S-48) were provided by the Institute of Parasitology, University of Veterinary Medicine, Vienna, Austria. Sporulated oocysts were obtained as previously described [16]. For the preparation of OLA, sporulated oocysts were further purified by sucrose density gradient (1.2 g/cm^3^) centrifugation (1200 x *g*, 20 min without break) modified from Dubey and colleagues [25] and Dumetre and Darde [26], washed and filtered (40 μm). Oocysts were then homogenised by three repeated freeze-thaw cycles in liquid nitrogen and subsequently mechanically disrupted (2 x 6500 rpm, 2 x 20 sec) using 2 ml tubes prefilled with 0.1 mm glass beads (Precellys glass kit, PeqLab, Germany) and a Precellys24 homogeniser device (Bertin Technologies, France). After the addition of protease inhibitor (cOmplete Mini Protease Inhibitor Cocktail Tablets, Roche Diagnostics, Mannheim, Germany) the suspension was centrifuged (1300 rpm/157 x g for 6 min) and OLA was collected and stored at -80°C until use.

Tachyzoites were derived from Vero cell cultures, filtered (5 μm) and further purified by discontinuous Percoll (GE Healthcare Biosciences AB, Uppsala, Sweden) density gradient centrifugation. Tachyzoite lysate antigen (TLA) was prepared by 3 freeze-thaw cycles using liquid nitrogen as previously described [16].

Protein concentration of OLA and TLA was tested by BCA protein assay as per protocol (Thermo Scientific, Waltham, MA, USA). Endotoxin levels were determined with an endpoint chromogenic Limulus Amoebocyte Lysate assay (LAL Assays, Lonza, Walkersville, MD, USA). Both antigen lysates (OLA and TLA) were characterized by SDS-PAGE and Western-blot and stored at -80°C until use.

### Splenocyte and dendritic cell stimulation assays and cytokine detection

To characterise the immunostimulatory profile of OLA *in vitro*, OLA was used to stimulate splenocytes and bone marrow-derived dendritic cells (BMDC), obtained from naive BALB/c mice. Bone marrow precursor cells, extracted from murine femurs and tibias as previously described [27], were cultivated with granulocyte-macrophage colony-stimulating factor (GM-CSF 20ng/ml, Sigma Aldrich, St. Louis, MO, USA) in complete medium (RPMI1640 supplemented with 10% heat-inactivated FCS, FCS, 2 mM L-glutamin, 2 mM mercaptoethanol, 100 μg/ml gentamicin, Sigma Aldrich, Saint Louis, MO, USA) for eight days. Single cell suspensions from spleens (5 x 10^6^/ml) and BMDC (1 x 10^6^/ml) were incubated with OLA (5 μg/ml and 50 μg/ml, respectively). Prior to use, OLA was preincubated with polymyxin B (Sigma-Aldrich) to inactivate possible LPS contamination as described previously [28]. Supernatants were collected after 24 h from BMDC cultures and after 48 h from splenocyte cultures to determine IFN-γ, IL-6 and IL-10 levels by ELISA (eBiocience).

### Stimulation of TLR-transfected HEK cells *in vitro*

Human embryonic kidney (HEK293) cells stably transfected with TLR2, TLR4 or TLR9 were kindly provided by Maria Yadzdanbakhsh (Department of Parasitology, Leiden University, Leiden, The Netherlands), Barbara Bohle (Institute of Pathophysiology, Medical University of Vienna, Vienna, Austria) and Martin Schwarzer (Laboratory of Gnotobiology, Institute of Microbiology of the Czech Academy of Sciences, Novy Hradek, Czech Republic) respectively. Plated HEK293 cells (5 x 10^5^/ml) were stimulated with OLA/polymyxin B (1, 10 and 100 μg/ml) and the respective controls Pam3Cys (1 μg/ml), LPS (2 ng/ml), CpG (100 μg/ml) or medium/polymyxin B for 24 h. Levels of human IL-8 were then measured in supernatants by ELISA (eBioscience).

### Experimental design and immunisations

**Fig 1:**
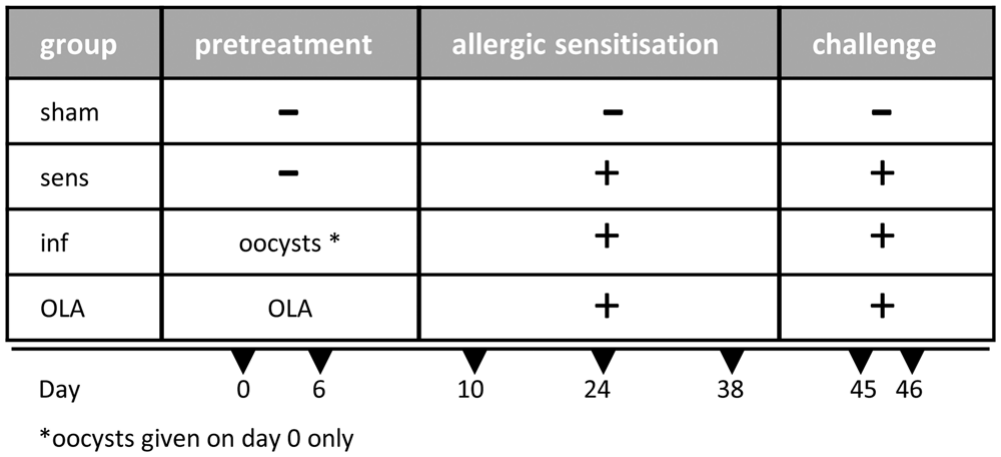
Treatment schedule. Mice were either infected orally with *T*. *gondii* oocysts on day 0 or immunised intraperitoneally with the oocyst lysate antigen OLA on day 0 and day 6. Sensitised controls and sham controls received GERBU/PBS on day 0 and day 6. Thereafter *T*. *gondii*-infected, OLA-pretreated and sensitised control mice were sensitised by three subcutaneous injections with the major birch pollen allergen r Bet v 1/Alum on day 10, 24 and 38 followed by an aerosol challenge with birch pollen extract on days 45 and 46. Sham controls received PBS/Alum on day 10, 24 and 38 and PBS on day 45 and 46. The experiment was terminated on day 49.

BALB/c mice were pretreated intraperitoneally on day 0 and day 6 with OLA (35 μg) preincubated with polymyxin B (Sigma) and thereafter adsorbed to 50 μl of GERBU, a better tolerated new generation of Freund’s complete adjuvant (GERBU Biotechnik, Heidelberg, Germany) (Fig 1). In order to infect mice, 500 *T*. *gondii* oocysts were inoculated in 300 μl of 3% NaHCO_3_ via gavage on day 0. Sham-immunised mice received PBS/polymyxin B mixed with GERBU on day 0 and 6. Thereafter, all mice were sensitised subcutaneously three times with 1μg recombinant Bet v 1 (rBet v 1, Biomay GmbH, Vienna, Austria) absorbed to 100μl aluminium hydroxide (Al(OH)_3_ containing 590–710 μg aluminium per 100 μl depending on the batch, Serva, Heidelberg, Germany) in two-week intervals (day 10, 24, 38). One week after the last sensitisation an aerosol challenge with 1% birch pollen extract (BP, Välinge, Sweden) was performed on two consecutive days (days 45 and 46) as previously described [29]. In short, mice were exposed to the aerosolised allergen suspension (De Vilbiss nebulizer 646, Somerset, PA, USA) for 20 min/day in an enclosed chamber. Sham-immunised and sham-sensitised mice received PBS/polymyxin B/GERBU on day 0 and 6, Al(OH)_3_/PBS on days 10, 24, 38 and an aerosol challenge with PBS on day 45 and 46. The experiment was terminated on day 49 when mice were terminally anaesthetised with isoflurane before bronchoalveolar lavages were performed. Spleens, lungs and bronchial lymph nodes (BLN) were removed and single cell suspensions were prepared by mechanical disruption for further analysis. The experiment was repeated three times.

### Brain cysts enumeration

Brains were extracted after terminal anaesthesia and homogenised in 1 ml of PBS using syringes with decreasing diameters before counting under a light microscope as previously described [16].

### Parasite- and allergen-specific antibody levels in sera

Blood samples were collected one day prior to the first treatment or infection and at the end of the experiment. Sera were stored at -20°C until quantification of *Toxoplasma-*specific and Bet v 1-specific antibodies as previously described [16]. The beta-hexosaminidase release assay was performed to determine the presence and functionality of Bet v 1-specific IgE antibodies and serves as an equivalent to allergic manifestation *in vivo* [30, 31]. Briefly, rat basophil leukaemia cells (RBL-2H3 cells) were incubated with diluted sera (1:300) before Bet v 1 (0.3 μg/ml) was added in Tyrode´s buffer to induce degranulation. Results represent the percentage of total β-hexosaminidase release after addition of 1% Triton X-100. In order to investigate *Toxoplasma-*specific antibody or recall responses, we previously established assays based on extracts derived from *T*. *gondii* tachyzoites [16]. Recent data from transcriptomic and proteomic analysis of *T*. *gondii* revealed that a high percentage of antigens is shared between the tachyzoite and oocyst stage [32, 33] thus TLA was used to assess *Toxoplasma* -specific humoral and cellular immune responses in OLA-immunised and infected mice. Results are expressed in optical density (*Toxoplasma*-specific), ELISA units (Bet v 1-specific) or as percentage of total beta-hexosaminidase release.

### Cytokine analysis *in vitro* in spleen and lung cell cultures

Lung cells and splenocytes (1 x 10^6^/100 μl) were incubated with either medium alone, TLA (50 μg/ml) or BP (50 μg/ml) at 37°C and 5% CO_2_ for 72 h before supernatants were removed. Cytokine levels of IFN-γ and IL-10 were quantified by ELISA in supernatants of splenocyte cultures whereas IL-4 and IL-5 was determined in supernatants from lung cell cultures (ready-Set-Go Kit, eBioscience, San Diego, CA, USA).

### Sampling of bronchoalveolar lavage fluid and lung tissue

At the end of the experiment bronchoalveolar lavage fluids (BALF) were collected. Cytospins of BALF cells were performed and further stained with haematoxylin and eosin (H&E; Hemacolor, Merck, Germany) for differential counts as previously described [20]. Lung tissue samples were fixed in 10% formaldehyde-PBS after bronchoalveolar lavages. Subsequently tissue samples were embedded in paraffin and stained with H&E and PAS. A histopathological scoring of the lung tissue sections was performed by evaluating the following parameters: PAS positive stained cells in bronchoalveolar lumina, lymphocyte infiltration surrounding bronchi and blood vessels, vascular hypertrophy and thickening of alveolar septa (see S1 Table adapted from [34]).

### Flow cytometry analysis

For the analysis of regulatory T cells, splenocytes were stained with the mouse regulatory T cell staining kit according to the manufacturer’s protocol (Mouse regulatory T cell staining Kit, CD4-FITC, CD25-PE, Foxp3-APC, eBioscience). Myeloid-derived suppressor cells (MDSC) were identified in lung cells after incubation with purified anti-mouse CD16/32 antibody followed by labelling with 7AAD (10 ng/ml; Sigma), CD11b-PerCP-Cy5.5, Ly6G-FITC, and Ly6C-APC or the relevant isotype controls. Two phenotypes of MDSCs (CD11b^+^Gr1^+^) were distinguished by flow cytometry i.e. the granulocytic (G-MDSC) (CD11b^+^Ly6G^+^Ly6C^low^) and monocytic (M-MDSC) (CD11b^+^Ly6G^-^Ly6C^hi^) MDSC as previously described [35]. BMDC were collected after *in vitro* stimulation with OLA and stained with CD11c-FITC and CD40-PE (BD Pharmingen, San Jose, CA, USA), CD80-PerCP-Cy5.5 (BD Pharmingen), CD86-PE (BD Pharmingen) and MHCII-APC. Stained cells were analysed using a FACS Calibur flow cytometer (BD Biosciences, San Jose, CA, USA) and FlowJo (Tree Star, Ashland, OR, USA) Version 10 software. MDSC were evaluated following the gating strategy described by Waight et. al [36]. Unless stated otherwise, antibodies were purchased from eBioscience.

### Real-time reverse transcriptase PCR

At the end of the experiment total RNA was isolated from pooled BLN cells. After reverse-transcription of RNA into cDNA the expression of IL-10, TGF-β, Foxp3 and the house keeping gene ALAS mRNA was determined by RT-PCR as previously described [16].

### Statistics

Data were statistically analysed by two-way ANOVA. Group served as a fixed factor and experiment as a random factor. Comparisons either against sham group (for *Toxoplasma*-specific immune response) or against sensitized for treatment effects were performed by linear contrasts. Residuals were tested for deviation from normality by Kolmogorov-Smirnov tests with Lilliefors’ corrected *p*-values, homogeneity of variances was tested by Levine’s tests. Groups were considered significantly different if the two-sided *p* value obtained in the pair wise test was below 0.05. All the statistical analyses were performed using Statistica 10.0 software (Statsoft, Tulsa, OK, USA).

## Results

### Inactivated oocysts (OLA) induced immunomodulatory cytokines in splenocytes and dendritic cells *in vitro*

**Fig 2:**
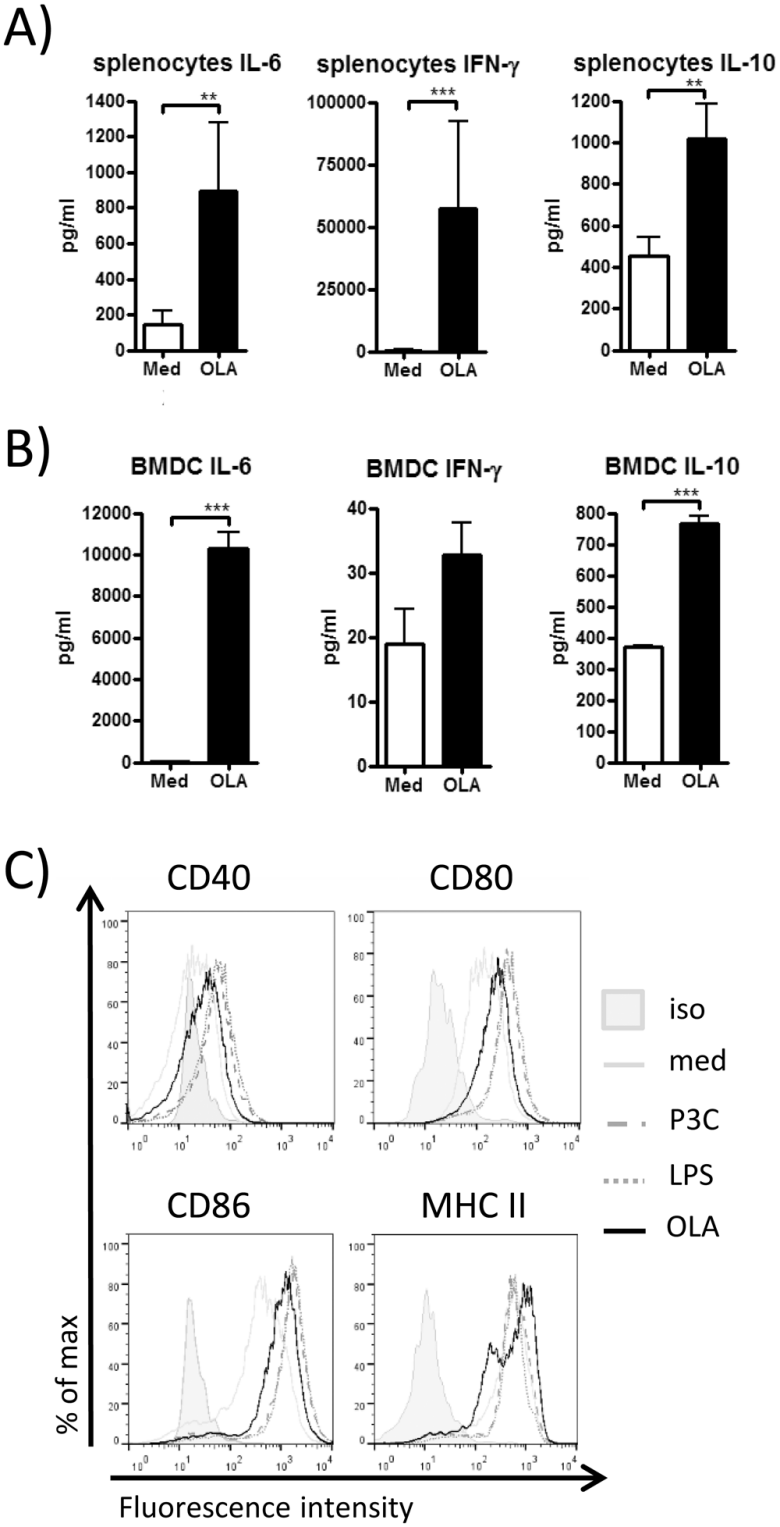
Cytokine production in splenocytes and BMDC and maturation of BMDC. Splenocytes (A) and BMDC (B) derived from naive BALB/c mice were stimulated with OLA or cultured with medium alone. IFN-γ, IL-6 and IL-10 levels were determined in supernatants by ELISA. Results show pooled values ± SEM of three to four independent experiments performed with cell suspensions prepared from one individual mouse per experiment. * *p* < 0.05; ** *p* < 0.01; *** *p* < 0.001. BMDC cultured with Pam3Cys, LPS, OLA or medium alone were analysed by flow cytometry for the expression of the maturation markers CD40, CD80, CD86 and MHCII after gating on CD11c^+^ cells (C). Representative histograms from one out of three repeated experiments.

We first tested the immunostimulatory capacity of OLA *in vitro* by stimulating splenocytes (Fig 2A) and BMDC (Fig 2B) derived from naive mice with the extract. In splenocytes, significant levels of IL-6, IFN-γ and IL-10 were produced upon stimulation with OLA compared to medium (Fig 2A). Likewise, incubation of BMDC with OLA led to a rise of IL-6, IFN-γ and IL-10 production in comparison to medium controls (Fig 2B). Additional flow cytometry staining for maturation markers showed that CD40, CD80, CD86 and MHCII levels were increased in BMDC cultured with OLA (Fig 2C).

### OLA is recognized by TLR2

**Fig 3:**
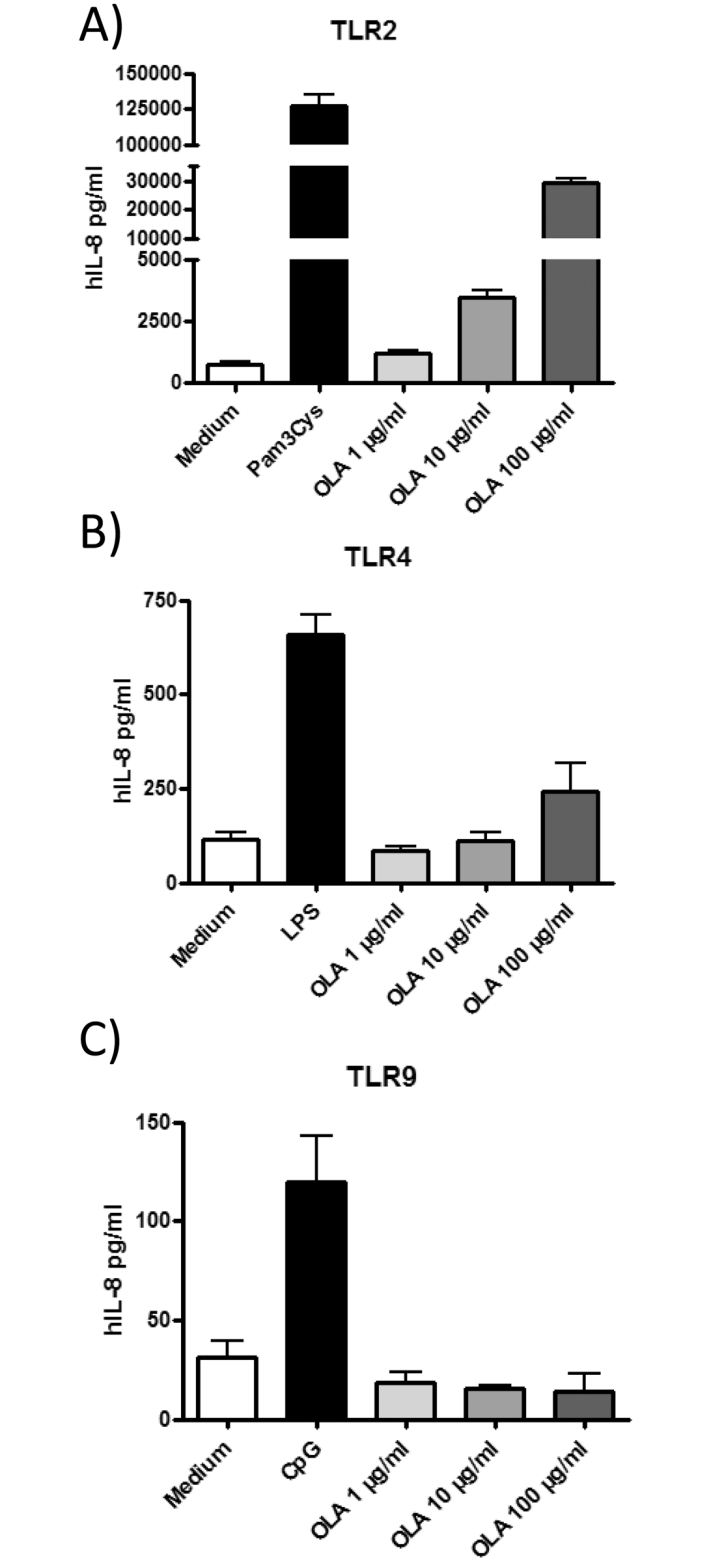
TLR activation by OLA. TLR2- (A), TLR4- (B) and TLR9- (C) transfected human embryonic kidney cells (HEK293) cells were incubated with OLA at three different concentrations and the respective positive controls (Pam3Cys, LPS and CpG) for 24h. Activation was assessed by measuring human (h)IL-8 production by ELISA. Results represent mean values ± SEM from one representative experiment.

To test whether OLA can signal through TLRs and thereby stimulate cytokine production, TLR-transfected HEK293 cells were incubated with OLA resulting in a dose dependent activation of TLR2 (Fig 3A) and limited activation of TLR4 at the highest concentration but not of TLR9 (Fig 3B and 3C).

### Immunisation with OLA induced *Toxoplasma*-specific humoral and cellular immune responses *in vivo*

**Fig 4:**
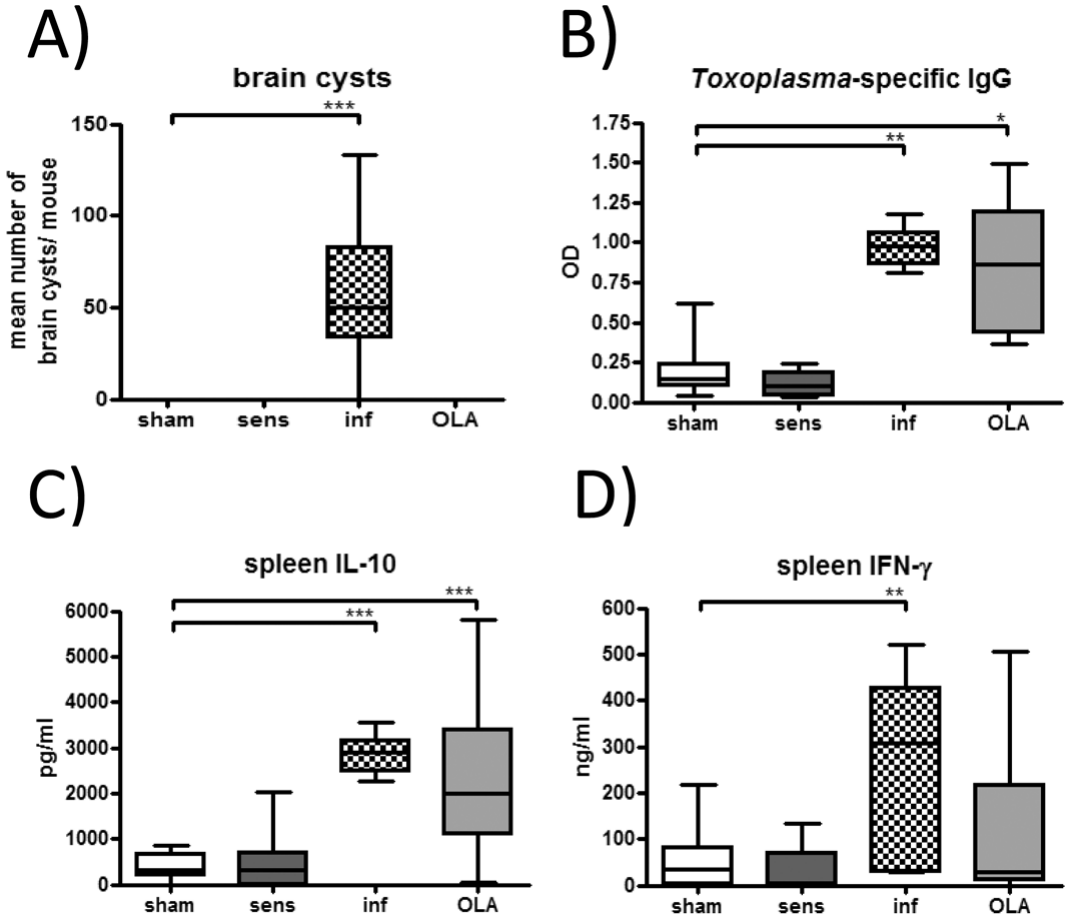
Brain cyst counts and parasite-specific antibody and cytokine production. Brain cysts were counted on day 49 (A). Results represent pooled values from two independent experiments with four to eight mice per group. *Toxoplasma*-specific IgG antibodies were measured in sera (B). IL-10 (C) and IFN-γ (D) production was determined in *Toxoplasma*-stimulated splenocytes *in vitro*. Spleens and sera were sampled on day 49. Results represent pooled values from three independent experiments with four to eight mice per group. * *p* < 0.05; ** *p* < 0.01; *** *p* < 0.001.

First, we confirmed that OLA, prepared from live parasite oocysts, did not contain viable parasites as demonstrated by the absence of brain cysts in OLA-pretreated mice (see Fig 1 for the treatment schedule). Cysts were only present in brains of infected mice (Fig 4A). To test whether OLA can induce parasite-specific humoral and cellular immune responses, levels of antibodies and cytokines were measured in OLA-immunised mice. Importantly, immunisation with OLA induced significant levels of *Toxoplasma*-specific IgG in sera and led to increased levels of IL-10 in spleen cell cultures after *Toxoplasma*-specific stimulation (Fig 4B and 4C). IFN-γ levels were also increased, though not significantly, in restimulated splenocytes of OLA-immunised mice compared to sham-immunised mice (Fig 4D). These results are comparable to those observed after infection where high levels of *Toxoplasma*-specific IgG in sera along with high levels of IFN-γ and IL-10 in restimulated splenocytes were measured (Fig 4B–4D).

### Pretreatment with OLA suppressed allergic airway inflammation in a comparable matter as infection with live *T*. *gondii* parasites

**Fig 5:**
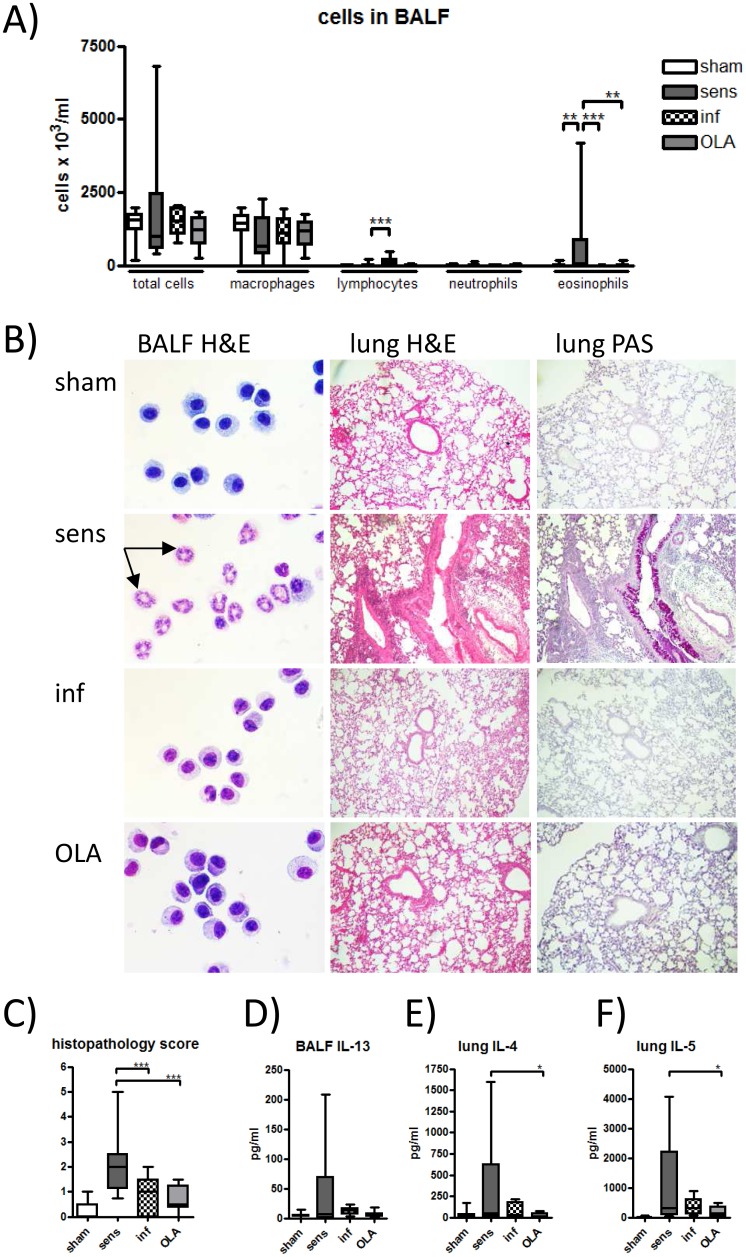
Allergic airway inflammation. Bronchoalveolar lavage fluids (BALF) were collected on day 49 and differential cell counts (total cells, macrophages, lymphocytes, neutrophils and eosinophils) were performed after H&E staining of cytospins (A). BALF cell counts represent pooled values from two independent experiments with five to eight mice per group. Representative H&E-stained BALF cells with arrows (↑) indicating eosinophils at a 100 x magnification from one representative experiment out of three (B). Representative lung tissue sections stained with H&E and Periodic Acid Schiff (PAS) at x 40 magnification. Lung histopathology scores were evaluated from two independent experiments and are presented as pooled values (C). IL-13 (D) levels were determined by ELISA in BALF sampled from two independent experiments with four to eight mice. IL-4 (E) and IL-5 (F) production was measured in supernatants of birch pollen restimulated lung cells by ELISA from three independent experiments with four to eight mice. Results represent pooled values. * *p* < 0.05; ** *p* < 0.01; *** *p* < 0.001.

The capacity of OLA pretreatment to modulate allergic lung inflammation was tested by evaluating the BALF cell composition and histological changes in the lungs of birch pollen sensitised and challenged mice. Pretreatment with OLA, as infection with *T*. *gondii*, resulted in a significant reduction of eosinophils in BALF compared to sensitised controls (Fig 5A). In OLA-pretreated mice the lymphocytes in BALF were as low as in the sham-treated or sensitised controls. In contrast, the inhibition of eosinophil counts in infected mice was accompanied by an increase of the lymphocyte population (Fig 5A). Furthermore, in OLA-pretreated mice reduced cellular infiltrates, mucus production and goblet cell hyperplasia in the H&E and PAS stained lung sections were detected compared to sensitised controls as indicated by the histological pathology score of the lungs (Fig 5B and 5C).

### Pretreatment with OLA reduced allergen-specific cellular and humoral immune responses

**Fig 6:**
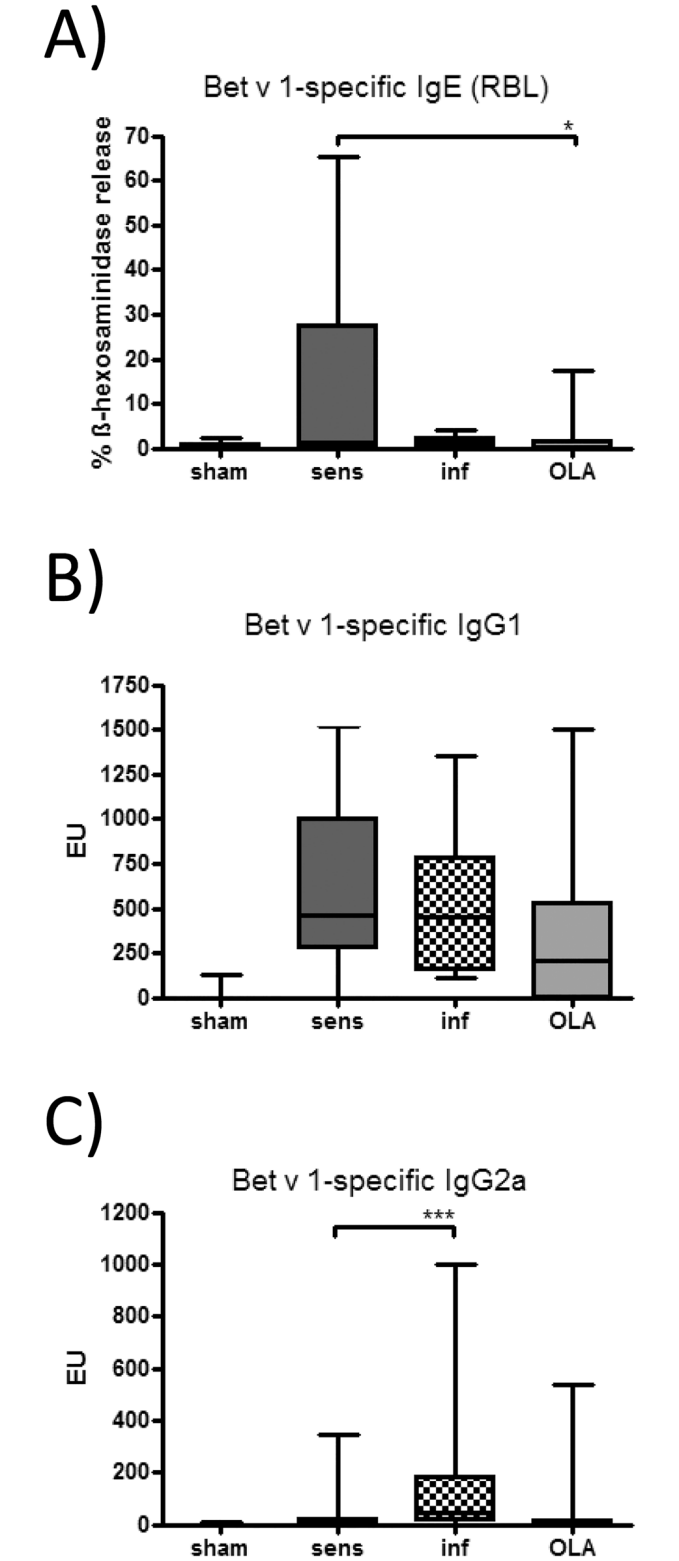
Allergen-specific antibody levels. The release of functional Bet v 1-specific IgE was quantified by β-hexosaminidase release from rat basophil leukaemia cells (RBL) (A). Bet v 1-specific IgG1 (B) and IgG2a (C) antibody levels were measured by ELISA. Antibody isotypes were determined in sera collected on day 49. Results represent pooled values from three independent experiments with four to eight mice per group. * *p* < 0.05; *** *p* < 0.001.

Along with the reduced eosinophilic airway inflammation, IL-13 levels in BALF tended to be lower in OLA-pretreated mice and the production of IL-4 and IL-5 was significantly reduced in BP-restimulated lung cells of OLA-pretreated mice compared to sensitised controls (Fig 5E and F). In accordance, pretreatment with OLA significantly prevented Bet v 1-specific IgE-dependent basophil degranulation compared to the sensitised controls (Fig 6A). Allergen-specific IgG1 levels followed the same trend (Fig 6B). Similar changes were detected in the mice infected before sensitisation. Allergen-specific IgG2a levels were at comparable levels in the OLA-pretreated mice and in the sensitised controls, although there was significant induction of IgG2a in the mice infected prior to sensitisation (Fig 6C).

### Pretreatment with OLA increased regulatory T cells in splenocytes and MDSC in lungs

**Fig 7:**
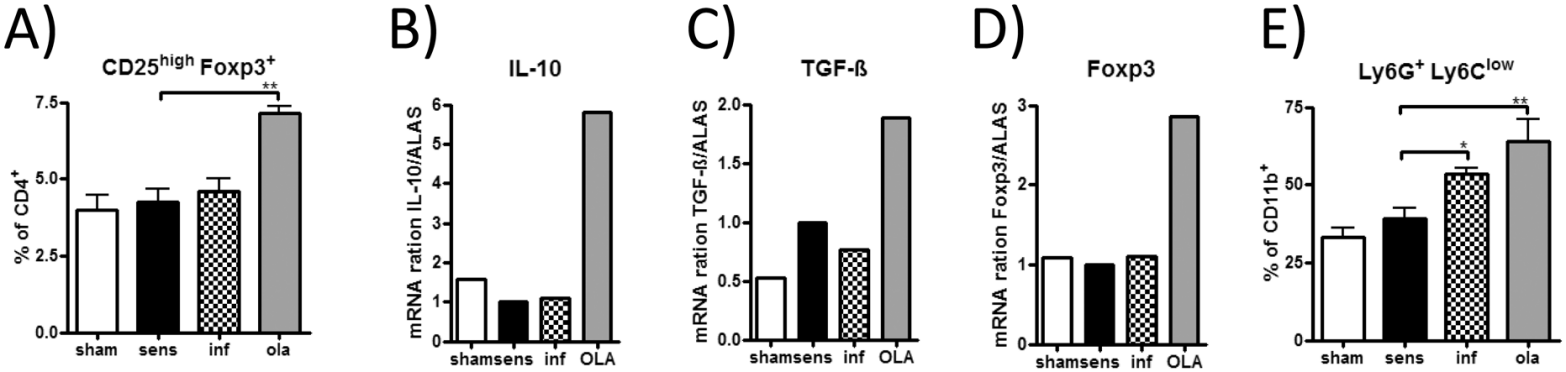
Regulatory T cells in spleen and myeloid-derived suppressor cells in lung. Splenocytes, lung and BLN cells were sampled on day 49. Regulatory T cells were identified as CD4^+^CD25^high^Foxp3^+^ lymphocytes in spleen cell suspension by flow cytometry analysis (A). Results represent values from one representative experiment with seven to eight mice per group. Expression of IL-10, TGF-β and Foxp3 was measured in pooled BLN cell samples by real-time RT-PCR and is presented as relative ratio to the house keeping gene ALAS (B-D). Results represent data from one representative experiment. G-MDSC (CD11b^+^Ly6G^+^Ly6C^low^) were identified by gating on live (7AAD^-^) CD11b^+^ lung cells by flow cytometry analysis (E). Results represent pooled values from two experiments with four to eight mice per group. * *p* < 0.05; ** *p* < 0.01.

We tested whether pretreatment with OLA had an influence on the regulatory T cell subset in spleens. Indeed, pretreatment with OLA led to an increase of CD4^+^CD25^high^Foxp3^+^ regulatory T cells when compared to sensitised controls (Fig 7A). Furthermore, also locally in bronchial lymph nodes the mRNA expression of the regulatory cytokines IL-10 and TGF-ß and the transcription factor Foxp3 was markedly increased in OLA-pretreated mice (Fig 7B–7D).

It has been shown previously that *T*. *gondii* infection increased numbers of MDSC in lungs [37]. Thus, we analysed whether not only *T*. *gondii* infection but also pretreatment with OLA leads to induction of this cell population. Indeed, infected and even more so OLA-pretreated mice displayed an increased percentage of the G-MDSC (CD11b^+^Ly6G^+^Ly6C^low^) subset in lungs when compared to sensitised controls (Fig 7E).

## Discussion

Epidemiologic studies described a lower prevalence of allergic diseases in individuals seropositive for *T*. *gondii* [10–14]. Previously, we provided experimental evidence that infection with *T*. *gondii* oocysts prevents allergic airway inflammation in a mouse model of birch pollen allergy [16]. Due to the obvious risks associated with infection, live pathogens are not suitable for the development of new strategies to reduce allergies in humans. Rather, the use of parasite-derived extracts or molecules is promoted. Accordingly, in the present study we now demonstrated that a parasite extract derived from *T*. *gondii* oocysts (OLA) successfully suppresses allergic airway inflammation, indicating that immunomodulation is not dependent on infection with viable oocysts.

First we characterised the immunostimulatory capacity of OLA on naive splenocytes and BMDC, key cells for antigen presentation and immunomodulation [38, 39]. This resulted in Th1 (IL-6 and IFN-γ) as well as regulatory (IL-10) cytokine production. Furthermore, stimulation with OLA led to maturation of BMDCs as indicated by the upregulation of MHCII and costimulatory molecules CD40, CD80 and CD86. Based on the results that OLA can activate BMDC, thereby stimulating Th1 and regulatory cytokine production in these cells, we suggest that the extract may also exhibit immunomodulatory properties and mediate suppression of allergic immune responses *in vivo*.

To evaluate this hypothesis we compared application of the extract OLA to infection with the viable oocysts *in vivo*. Indeed, immunisation with OLA led to the production of *Toxoplasma*-specific IgG antibodies along with Th1 and regulatory cytokines comparable to the infected controls and as previously shown in our model of *T*. *gondii* infection [16]. Regarding the immunomodulatory capacity of the oocyst extract, the key question was whether OLA can mediate protection against allergic airway inflammation in our model of birch pollen allergy in a way similar as *T*. *gondii* infection does. Importantly, pretreatment with OLA significantly reduced the influx of eosinophils, peribronchial and perivascular infiltration of inflammatory cells including lymphocytes as well as mucus production in lungs. These findings are relevant since histological changes such as the infiltration of inflammatory cells with an accumulation of eosinophils are characteristically provoked in sensitised mice after an aerosol challenge [40] and correlate with increased airway hyperreactivity, a typical clinical feature of asthma [41]. The fact that infection with *T*. *gondii*, but not OLA, also led to an increase of lymphocytes in BALF is most likely due to parasite replication which has been confirmed earlier to occur also in lung cells [42, 43]. Therefore these data support the concept of using parasite derived extracts for immunomodulation in order to avoid lung inflammation.

Moreover, in OLA-pretreated mice Bet v 1-specific IgE antibody levels were reduced, pointing out that allergic sensitisation in addition to airway inflammation can be effectively modulated by OLA. A clear shift from Th2- to Th1-related allergen-specific IgG2a antibodies along with a prominent induction of Th1-cytokines as described before in *T*. *gondii*-infected mice [16, 44], could however, not be detected after immunisation with OLA. It is therefore assumed that regulatory mechanisms rather than a Th1/Th2 bias seem to be involved in the suppression of the allergic immune response with OLA.

Regulatory T cells are induced following certain parasitic infections to limit host pathology and at the same time facilitate parasite survival in the host [45–47]. Our finding that preferentially application of OLA led to an increased percentage of CD4^+^CD25^high^Foxp3^+^ regulatory T cells in spleens extend on our previous results showing an increase in regulatory T cells in *Toxoplasma*-infected mice [16]. These findings are of importance as parasite-induced regulatory T cells have been shown to lead to suppression of immune responses towards unrelated antigens such as allergens in murine asthma models [48, 49]. Here we also report an increase of Foxp3 expression in lung draining bronchial lymph node cells of OLA-pretreated mice. The expansion of regulatory T cells in thoracic lymph nodes after infection with *T*. *gondii* was recently described, also showing that adoptive transfer of these cells resulted in suppression of allergic airway inflammation [17, 18].

The induction and survival of regulatory T cells has been demonstrated to involve TLR2 signalling in mice [50, 51]. TLR can recognise a large variety of microbial patterns [39] and constitute an important pathway of parasite interaction with the innate immune system. With regard to *T*. *gondii* infection we previously described the upregulated expression of TLR2, 4, 9 and 11 in splenocytes [16]. Here we describe that OLA was mainly recognised by TLR2. In line with our data it has been reported that TLR2 agonists can modulate allergic immune responses [52].

Apart from the regulatory T cells, MDSC gained increasing attention for their potential to regulate innate and adaptive immune responses within the last years [53]. Both subsets of the MDSC, the G-MDSC and M-MDSC [35], have been described to supress T cell proliferation *in vitro* [54]. In accordance with data by Voisin et. al [37], MDSC were augmented in the lungs of *T*. *gondii* infected animals compared to uninfected controls. Notably we here show that not only infection but even to a greater extend the application of OLA led to an expansion of the G-MDSC. Moreover, data by Song et al. [55] showed that the transfer of MDSC could inhibit allergic airway inflammation, suggesting a possible role of MDSC in allergy suppression in our model. The actual regulatory capacity of these cells need further evaluation in respective suppression assays and transfer experiments.

Taken together, our data show that OLA, an antigen extract derived from *T*. *gondii* oocysts, possesses strong modulatory properties *in vitro* and *in vivo*. Pretreatment with OLA prevented allergic sensitisation and airway inflammation. This phenomenon was accompanied by the induction of regulatory T cells in spleens and BLN as well as G-MDSC in lungs. These two regulatory cell populations might act together and contribute to the allergy prevention. The future aim is to identify those molecules within OLA that exhibit the anti-allergic potential and intrinsic adjuvant properties. It will be important to further evaluate the immunomodulatory function and safety profile before translation of such new immunomodulators/adjuvant systems into the clinics for the next generation of prophylactic and therapeutic strategies against allergies.

## Supporting Information

S1 Table Scoring criteria for the histopathological assessment of lung tissue sections. (DOCX)
