# Supplementary material for: Oocyst-Derived Extract of Toxoplasma Gondii Serves as Potent Immunomodulator in a Mouse Model of Birch Pollen Allergy
Source: PLoS One. 2016 May 5;11(5):e0155081. doi: 10.1371/journal.pone.0155081 (PMC4857930; doi:10.1371/journal.pone.0155081)
Supplement: S1 Table — (DOCX) [file pone.0155081.s001.docx]

**S1 Table. Scoring criteria for the histopathological assessment of lung tissue sections.**

| **Severity** | **PAS positive stained cells per 100 counted bronchoalveolar epithelial cells** | **Lymphocyte infiltration around bronchi and blood vessels** | **Vascular hypertrophy** | **Thickening of alveolar septa** | **score** |
| --- | --- | --- | --- | --- | --- |
| **No change** | 0/100 | - | - | - | **0** |
| **Moderate** | ≤ 50/100 | Infiltrates less than 10 cells thick | Thickening without narrowing | Increased cellularity in septa | **1** |
| **Severe** | ≥ 51/100  and/or mucous plaque | Infiltrates 10 cells thick and above | Thickening and narrowed lumen | Continuous lining of cells in septa | **2** |
